# Involvement of p53 in the cytotoxic activity of the NAMPT inhibitor FK866 in myeloid leukemic cells

**DOI:** 10.1002/ijc.27726

**Published:** 2012-07-20

**Authors:** Basant Kumar Thakur, Tino Dittrich, Prakash Chandra, Annette Becker, Wolfgang Kuehnau, Jan-Henning Klusmann, Dirk Reinhardt, Karl Welte

**Affiliations:** 1 Department of Pediatric Hematology and Oncology, Hannover Medical SchoolCarl Neuberg Str-1, 30625 Hannover, Germany; 2 Department of Molecular Hematopoiesis, Hannover Medical SchoolCarl Neuberg Str-1, 30625 Hannover, Germany; 3 Frankfurt University Medical SchoolTheodor-Stern-Kai -7, 60590 Frankfurt, Germany; 4 Department of Biology, Technische Universität DarmstadtDarmstadt 64287, Germany; 5 Institute for Humangenetics, Hannover Medical SchoolCarl Neuberg Str-1, 30625 Hannover, Germany

**Keywords:** p53, acetylation, NAMPT, sirtuins (SIRTs), FK866, nicotinamide (NA)

## Abstract

FK866 is a specific inhibitor of NAMPT and induces apoptosis of leukemic cells by depletion of intracellular NAD^+^. Since up-regulation of NAMPT is associated with several cases of cancers, including leukemias, we asked whether in leukemic cells inhibition of NAMPT involves p53 pathway. We observed that FK866 induced apoptosis and reduced cell proliferation in NB-4, OCI-AML3 and MOLM-13 cell lines. In contrast, the leukemia cell lines, K-562 and Kasumi, containing nonfunctional p53 were relatively unaffected by FK866 treatment. Importantly, direct inhibition of sirtuins significantly reduced the viability of NB-4, OCI-AML3 and MOLM-13 cell lines. Activation of p53 by FK866 involved increased acetylation of p53 at lysine 382 with subsequent increase in the expression of *p21* and *BAX*. Further, knockdown of p53 attenuated the effects of FK866 on apoptosis and cell cycle arrest, which was partly associated with decreased expression of *p21* and *BAX*. Our results suggest the role of p53 acetylation pathway in the anti-leukemic effect of FK866.

In recent years, epigenetic changes have been associated with several types of cancer, including myelodysplastic syndrome (MDS) and acute myeloid leukemia (AML).^1^ Among all the described modifications, the acetylation of lysine residues located at the N-terminal tail of histones play a major role in regulating gene expression.^2^ Besides histones, several non-histone proteins, which include tumor suppressors, signaling molecules, receptors, chaperones and DNA repair enzymes are also regulated by acetylation.^3^ Histone deacetylases (HDACs) are a family of proteins that, in healthy cells, maintain normal acetylation levels of proteins. Impaired acetylation level, due to aberrant HDAC activity, is often related to pathological malignancies. HDACs are classified into four classes: Class I, Class II, Class III, Class IV.^4^ Sirtuins (SIRTs) belong to Class III of HDACs family. They are distinct from the classical HDACs in that they require NAD^+^ to mediate the deacetylation reaction.^5^ The histone deacetylase inhibitors (HDACIs) are a new class of cytostatic agents known to be effective against different types of cancers, including leukemias.^6^ The possible mechanism underlying the action of HDACIs is induced acetylation of target proteins leading to the correction of the gene expression pathway in leukemic cells.

Recently, SIRTs have emerged as important HDACs which coordinate complex gene expression programs through deacetylation of target proteins involved in cellular metabolism and homeostasis.^7^–^9^ The strict requirement for NAD^+^ by SIRTs to perform deacetylation reaction, in turn, makes SIRT1 highly dependent on the activity of NAMPT, a rate limiting enzyme in the NAD^+^ biosynthesis. It converts nicotinamide (NA) into nicotinamide mononucleotide (NMN), which is further converted into nicotinamide adenine dinucleotide (NAD^+^) by nicotinamide mononucleotide adenylyltransferase (NMNAT).^10^ In contrast to the prevalence of p53 mutations in solid tumors, only 10% of hematological malignancies contain mutant p53.^11^ This gives rise to the important question of how malignancies, like leukemia, develop in the presence of p53, a strong tumor suppressor protein. Inactivation of p53-mediated downstream signaling in such a situation can occur either by epigenetic modification or by abolished interaction of p53 protein with downstream effectors.^12^, ^13^ Acetylation of p53 is an important post-translational modification that allows p53 to induce the expression of genes relevant to tumor suppression.^14^ Activation and/or overexpression of SIRT1 has been linked with several cases of cancers, including Chronic Myelogeneous Leukemia (CML).^15^, ^16^ Since p53 is one of the major non-histone targets of SIRT1, it is considered that inhibition of p53 tumor suppressor function might be involved in cancer associated with hyperactivated SIRT1. Indeed, it has been recently reported that in cases of CML inhibition of SIRT1 by NA results in p53 dependent decrease in cell proliferation.^16^ A thorough understanding of the pathway involved in the regulation of p53 activity will be extremely useful in the development of new strategies for treatment of cancer, including restoration of p53 function and selective killing of tumors with wild type or mutant p53.

**What's new?**

While the enzyme inhibitor FK866 is known to have anti-tumor activity against various human cancer cells, researchers haven't fully understood how or why it works. The authors found that FK866 is able to induce apoptosis in leukemic cells by indirectly activating the tumor-suppressor protein p53. FK866 might, therefore, be useful in combination with existing chemotherapies in the treatment of p53-positive leukemias, and possibly also in drug-resistant leukemias that overexpress p53 inhibitors such as the sirtuin SIRT1.

Unlike other HDACs, little is known about the mechanism of inhibition of SIRTs, and the drugs that inhibit SIRT directly, or indirectly *via* inhibition of NAMPT pathway, are under development. NA, a noncompetitive inhibitor of SIRT at high concentration, is a compound shown to possess anti-tumor activity on several cancer cells *in vitro*.^16^, ^17^ FK866, a specific inhibitor of NAMPT has been shown to possess anti-tumor activity against various human leukemic cells, including AML.^18^–^20^ In contrast, the normal hematopoietic progenitor cells remain unaffected.^18^, ^20^ This might be explained by the fact that the metabolic rate of cancer cells is abnormally high, and therefore, they require higher NAD^+^ levels. As a consequence of this, cancer cells have an increased sensitivity toward low levels of the cellular NAD^+^ content.

The cytotoxic effect of FK866 on cancer cells is known, as indicated earlier. However, the knowledge on the molecular events involved in FK866-mediated death of leukemic cells is lacking. In the current study, we report the role of p53 acetylation in FK866-mediated death of leukemia cells and provide a possible link between p53 and the metabolic state of leukemia cells.

## Material and Methods

### Cell lines

NB-4 (ACC207), OCI-AML3 (ACC 582), MOLM-13 (ACC 554), K-562 (ACC 10) and Kasumi (ACC 220) cell lines were obtained from DSMZ (Braunschweig/Germany) and were cultured in media according to recommendations of DSMZ at 37°C in a humidified atmosphere of 5% CO_2_. RPMI 1640 (PAA, Pasching/Austria) and alpha MEM (GIBCO, Darmstadt/Germany) were supplemented with 1% l-glutamine, 1% penicillin and 1% streptomycin and appropriate amount of fetal calf serum (FCS) (PAA, Pasching/Austria). Cells were passaged every 2–3 days as mentioned in the datasheet from DSMZ.

### Knockdown of p53

For knockdown of p53 in NB-4 cells, shp53 lentiviral (sc-45917-V) and control lentiviral (sc-108080) particles were purchased from Santa Cruz Technology (Nogales/Arizona), and NB-4 cells were transduced and selected according to manufacturer's instructions. To perform the knockdown of p53 in OCI-AML3 and MOLM-13 cell lines, viral particles were produced by transient transfection of HEK 293T cells with target vector (Shp53-sense 5′- GATCCCCGGCACAGAGGAAGAGAATC TTTCAAGAGAAGATTCTCTTCCTCTGTGCTTTTTGGAA A-3′; Shp53-antisense 5′-AGCTTTTCCAAAAAGCACA GAGGAAGAGAATCTTCTCTTGAAAGATTCTCTTCCTC TGTGCCGGG-3′); oligonucleotide was obtained from Qiagen (Hilden/Germany) and cloned into pSUPER and further sub-cloned into pRRL.SF.DsYellow Ex.pre, pGagpol vector, pEnv-VSVG vector and REV vector. Cells (0.4 × 10^6^) were seeded in a 6-well plate (0.2 × 10^6^/ml) later transfected with calcium phosphate transfection method. Supernatant with viral particles was harvested after 36 hr. OCI-AML3 cells (0.1 × 10^6^) were seeded in a 24-well plate (0.2 × 10^6^/ml) and 50 μl of virus supernatant and 5 μl of polybrene (2 μg/ml) were added. Cells were incubated for 1 hr in the incubator and afterwards centrifuged for 2 hr at 37°C and 700 × *g*. After 16 hr of incubation, the medium was replaced. Cells were grown for 2 days to overcome stress produced by infection with virus particles and polybrene. Thereafter, cells positive for green fluorescent protein (GFP) were sorted by flow cytometry and the sorted cells were cultured further for experiments. The expression of p53 RNA and protein was assayed with quantitative real time-polymerase chain reaction (qRT PCR) and Western blotting, respectively.

### Western blotting and intracellular antigen staining

Equal number of pelleted cells were washed with ice-cold phosphate-buffered saline (PBS), again pelleted and resuspended in 1.5× Lämmli buffer (15% glycerol, 3% sodium dodedcyl sulfate, 4.5% β-mercaptoethanol, 0.2% bromophenol blue in 100 mM Tris/HCl [pH 6.8]), subsequently incubated at 95°C for 5 min and centrifuged in a microcentrifuge for 5 min. Samples were separated in 10% sodium dodecyl sulfate-polyacrylamide gels and transferred onto nitrocellulose membranes (Amersham/GE Healthcare, Freiburg/Germany). The membranes were blocked with 5% non-fat dry milk-tris buffered saline/TWEEN 20 0,1% (TBS-T) (10 mM Tris/HCl [pH 8.0], 150 mM NaCl, 0.1% Tween 20) for 1 hr at room temperature and incubated with primary antibody in TBS-T for 1 hr at room temperature or overnight at 4°C. After washing four times for 5 min each in TBS-T, membranes were incubated with horseradish peroxidase-conjugated secondary antibody (Santa Cruz Technology, Nogales/Arizona) for 1 hr at room temperature. After washing four times for 5 min, protein bands were visualized through chemiluminescence reaction (Pierce/Thermo Fisher Scientific, Bonn/Germany) followed by exposure to X-ray film (Agfa, Munich/Germany).

To quantify the acetylation status of p53 by flow cytomtry, OCI-AML3 cells were seeded and treated as for the apoptosis assay and stained using IntraPrep™ Permeabilization Reagent (BeckmanCoulter, Krefeld/Germany) and Alexa Fluor 647 Mouse anti p53 acK382, or respective isotype control antibody (BD Bioscience, Heidelberg/Germany). All assays were carried out according to manufacturer's instructions and stained cells were analyzed with a Navios flow cytometer (BeckmanCoulter, Krefeld/Germany).

### Antibodies

Rabbit polyclonal antibody to p53 and rabbit polyclonal antibody to acetyl-p53-K382 were obtained from Cell Signalling Technology (Danvers/Massachusetts). Mouse monoclonal antibody to β-actin was purchased from Santa Cruz Technology (Nogales/Arizona). Antibodies to phospho-p53-S15 and antibodies to phospho-p53-S392 were obtained from Cell Signalling Technology (Danvers/Massachusetts).

### Quantitative real-time PCR (qRT PCR)

RNA from cells was isolated using RNeasy Micro Kit (Qiagen, Hilden/Germany). Reverse transcription was performed with 500 ng of RNA and reagents of Omniscript Reverse Transcription Kit, both according to the manufacturers' protocol. The relative mRNA levels of target genes were measured as triplicates using SYBR Green Master Mix (Applied Biosystems, Foster City/California) and the following oligonucleotides (Qiagen, Hilden/Germany): β-actin-sense, 5′-TTC CTG GGC ATG GAG TC-3′ and β-actin-antisense, 5′-AGG TCT TTG CGG ATG TC-3′; p21-sense, 5′-ATG TGT CCT GGT TCC CGT CCT-3′ and p21-antisense 5′-CAT TGT GGG AGG AGC TGT GA-3′; p53-sense, 5′-GTT CCG AGA GCT GAAT GAG G-3′ and p53-antisense, 5′-TTA TGG CGG GAG GTA GAC TG-3′; BAX-sense 5′-GTG TCT CAA GCG CAT CGG GGA C-3′ and BAX-antisense 5′-GAG GAG TCT CAC CCA ACC ACC CTG-3′. Data were normalized to the β-actin signal using the ΔΔ*Ct* method and results are shown as mean ± standard error.

### Cell cycle and apoptosis assay

For the cell cycle analysis, cells were incubated for 1 hr in the medium containing 10 μM BrdU. Cells were permeabilized, fixed and stained with anti-BrdU antibody and 7AAD using the BrdU Flow Kit (BD Pharmingen, Heidelberg/Germany) according to manufacturer's instructions. Apoptosis analysis was performed using the AnnexinV-APC Apoptosis Detection Kit (BD Pharmingen, Heidelberg/Germany) according to manufacturer's instructions. Flow cytometry measurements were performed on a Navios AW39150 (Beckman Coulter).

### Cell counts assay

Cells were seeded in 96-well plate at a density of 5,000 cells per well. After treatment with FK866 for indicated time points, absolute cell counts were quantified using trypan blue cell exclusion assay. All reactions were analyzed as triplicates in two independent experiments.

### Measurement of intracellular NAD^+^ and ATP

Cells (0.1 × 10^6^) were seeded in a 12-well plate (0.1 × 10^6^/ml) and treated for the indicated time points with FK866. From that suspension 100 μl were transferred into an opaque plate for measurement of ATP with CellTiter Glo Luminescent Cell Viability Assay (G7570; Promega, Mannheim/Germany) according to manufacturer's instructions. The remaining cells were washed once in ice cold PBS and pelleted. The pellet was then homogenized in NAD^+^ extraction buffer from the EnzyChrom NAD^+^/NADH Assay Kit (E2ND-100; Biotrend, Cologne/Germany). Measurements were performed according to manufacturer's instructions.

## Results

### Status of p53 in leukemia cell lines and their sensitivity to FK866

**Figure 1:**
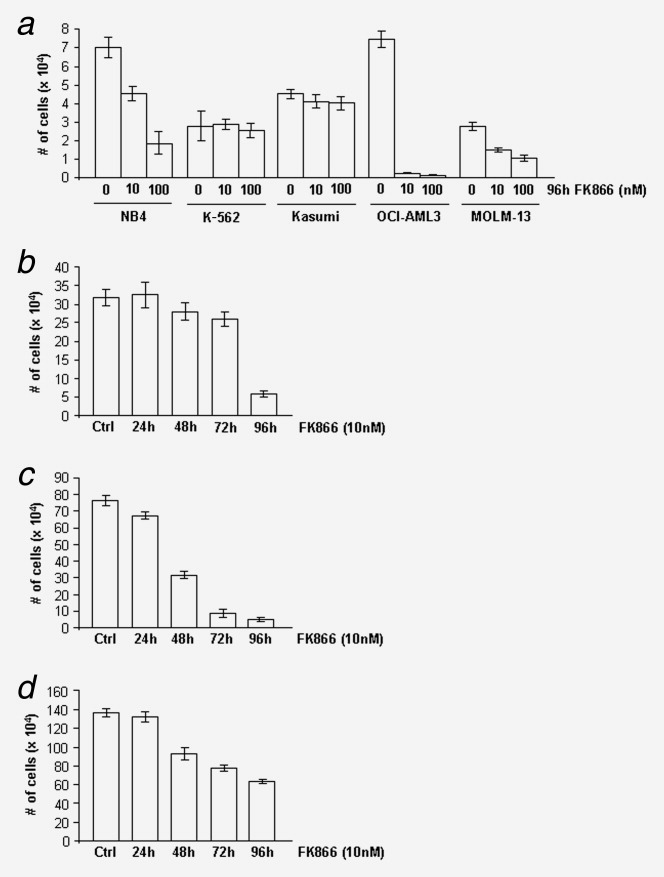
FK866-mediated inhibition of proliferation of leukemic cells. Cells were treated with the indicated concentrations of FK866 for different time points. Afterwards cell number was determined using trypan blue exclusion assay. (*a*) Five leukemic cell lines were exposed to different concentrations of FK866 for 96 hr. (*b*) NB-4 cells were incubated with 10 nM FK866 for indicated time points. (*c*) OCI-AML3 cells were incubated with 10 nM FK866 for indicated time points. (*d*) MOLM-13 cells were incubated with 10 nM FK866 for indicated time points.

FK866 is an inhibitor of NAMPT, an enzyme involved in the biosynthesis of the cofactor NAD^+^. The Class III HDACs, SIRT, require NAD^+^ to mediate deacetylation of their target proteins.^21^ Recently, we have shown that FK866 induces apoptosis and cell cycle arrest in NB-4 cells.^22^ In the current study, we selected a panel of cell lines (K-562, Kasumi, NB-4, OCI-AML3 and MOLM-13) based on different p53 status and compared their sensitivity toward FK866. K-562 cells carry a monoallelic insertion mutation in exon 5 resulting in a frameshift mutation and consequent expression of a truncated non-functional p53 protein of 148 amino acids. The Kasumi cell line in turn has a hot spot mutation in p53 (R248Q) which leads to almost complete abrogation of transcriptional activation. NB-4 cells carry a missense mutation (C176F) within p53 which interferes with its binding to certain target genes and attenuates their expression. In contrast, OCI-AML3 and MOLM-13 cells have wild type p53. We observed that NB-4, OCI-AML3 and MOLM-13 cell lines were highly sensitive to FK866 but, in contrast, K-562 and Kasumi cells were relatively resistant to FK866 treatment (Fig. 1*a*). Since NB-4, OCI-AML3 and MOLM-13 cells were sensitive to FK866 treatment, we further focused our study on these cell lines. Measurement of absolute cell counts for varying periods of treatment with FK866 (0, 24, 48, 72 and 96 hr) revealed that the absolute numbers of NB-4, OCI-AML3 and MOLM-13 cells were decreased. (Fig. 1*b-d*)

### FK866 induces apoptosis and cell cycle arrest in NB-4, OCI-AML3 and MOLM-13 cell lines

**Figure 2:**
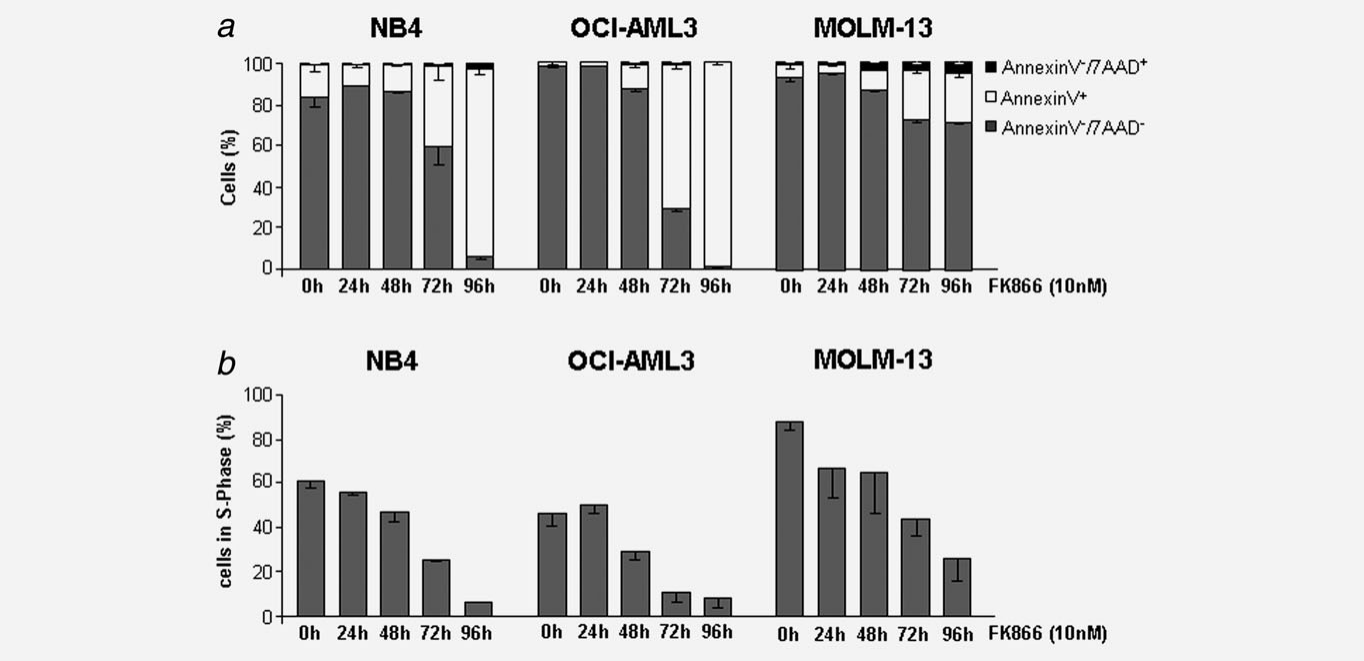
Percentage of cells undergoing apoptosis and cell cycle arrest increases upon exposure to FK866. (*a*) Percentage of AnnexinV^−^/7AAD^−^ (live), AnnexinV^+^ (apoptotic), AnnexinV^−^/7AAD^+^ (necrotic) was measured using flow cytometry in cell lines NB-4, in OCI-AML3 and in MOLM-13. (*b*) BrdU/7-AAD cell cycle analysis of NB-4 cells, of OCI-AML3 cells and of MOLM-13 cells. Diagram shows percentage of cells in S-phase.

In the presence of genotoxic stress, activation of p53 involves regulation of key cellular processes such as DNA repair, cell-cycle arrest, senescence and apoptosis.^23^ Because FK866 affected both, cell proliferation and viability, we performed AnnexinV/7-AAD staining and BrdU incorporation assay to measure apoptosis and cell cycle profile, respectively, after treating NB-4, OCI-AML3 and MOLM-13 cells for varying periods (0, 24, 48, 72 and 96 hr) with FK866. We found that in FK866 treated cells apoptosis increased (Fig. 2*a*) and the number of cells entering S-phase decreased (Fig. 2*b*) in time-dependent manner. Similar experiments with Kasumi cells harboring hot spot mutation in p53 showed no significant influence on cell cycle profile (Supporting Information Fig. 1A) and apoptosis (Supporting Information Fig. 1B).

### Cell death mediated by FK866 involves both depletion of intracellular NAD^+^ and ATP

**Figure 3:**
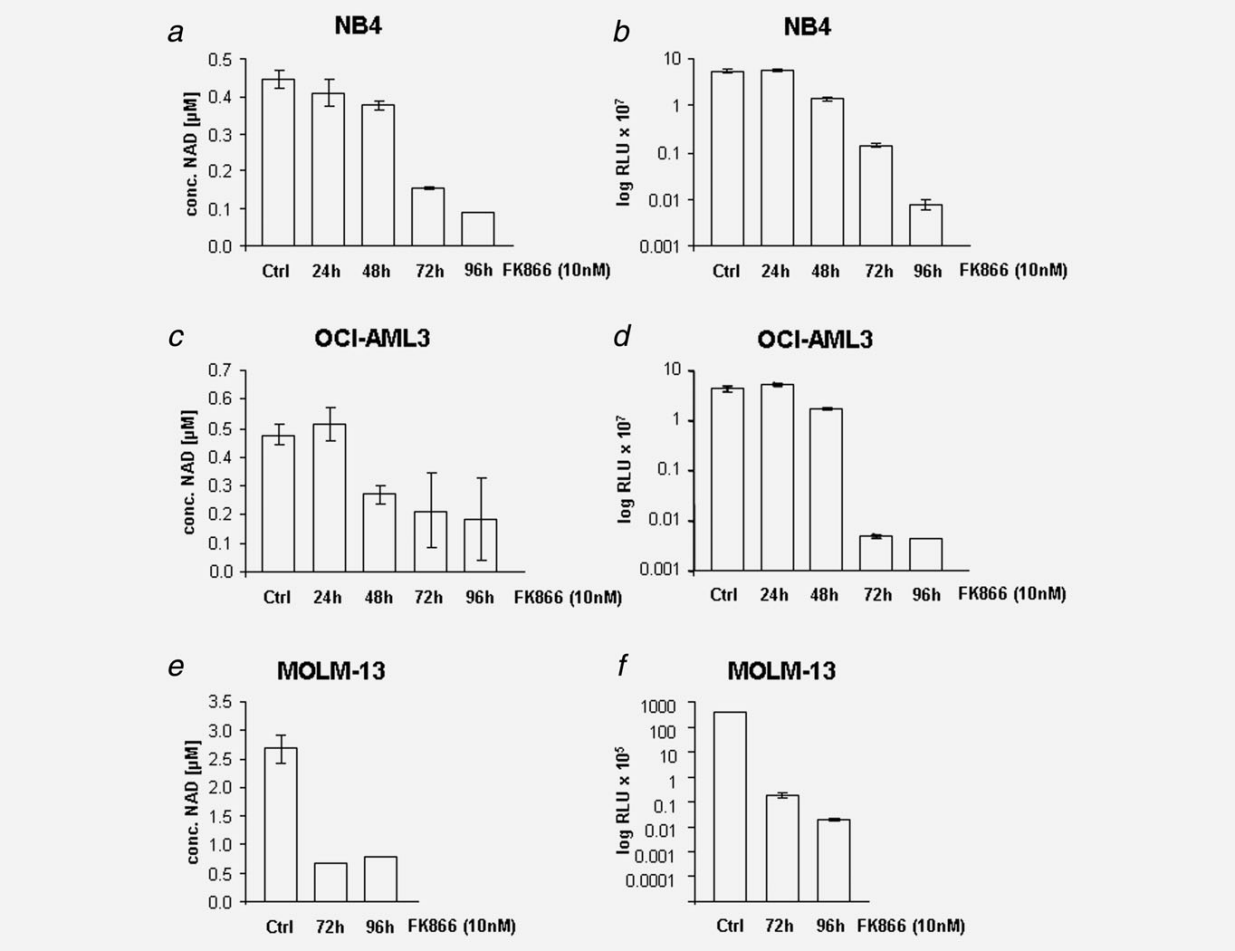
Treatment with FK866 results in gradual depletion of NAD^+^ followed by decreasing ATP levels. Cells were treated with 10 nM of FK866 for the indicated time points. (*a*) NAD^+^ levels were measured after exposure to FK866 in NB-4 cells, (*c*) in OCI-AML3 cells and (*e*) in MOLM-13 cells. (*b*) ATP levels were measured after exposure to FK866 in NB-4 cells, (*d*) in OCI-AML3 cells and (*f*) in MOLM-13 cells.

Mechanistically, FK866 induces cell death through NAD^+^ depletion which is followed by ATP depletion. Depletion of ATP correlates with cell death.^18^, ^24^, ^25^ Therefore, we evaluated the intracellular NAD^+^ and ATP content after FK866 treatment of NB-4 (Figs. 3*a* and 3*b*), OCI-AML3 (Figs. 3*c* and 3*d*) and MOLM-13 cells (Figs. 3*e* and 3*f*). In accordance to the previous reports, we also observed initial fall of NAD^+^ concentration followed by a decrease in ATP content in all the three cell lines. For MOLM-13 cells, we selected only two points, 72 hr and 96 hr, of FK866 exposure because we found that, in general, the effect of FK866 treatment on NAD^+^ and ATP was significantly observed at these time points. We further checked the NAD^+^ and ATP status in Kasumi and K-562 cells, the cell lines which were resistant to FK866. We found that the treatment of these cells with FK866 decreased the intracellular NAD^+^ (Supporting Information Fig. 2A) content similar to FK866 sensitive cell lines but, interestingly no significant change was observed when ATP concentrations (Supporting Information Fig. 2B) in these cells were measured. This indicates that FK866-mediated death in NB-4, OCI-AML3 and MOLM-13 cell lines requires depletion of intracellular ATP which follows after depletion of NAD^+^ in cell lines sensitive to FK866.

### Treatment with FK866 increases the acetylation levels of p53 and increases the expression of *p21* and *BAX*

**Figure 4:**
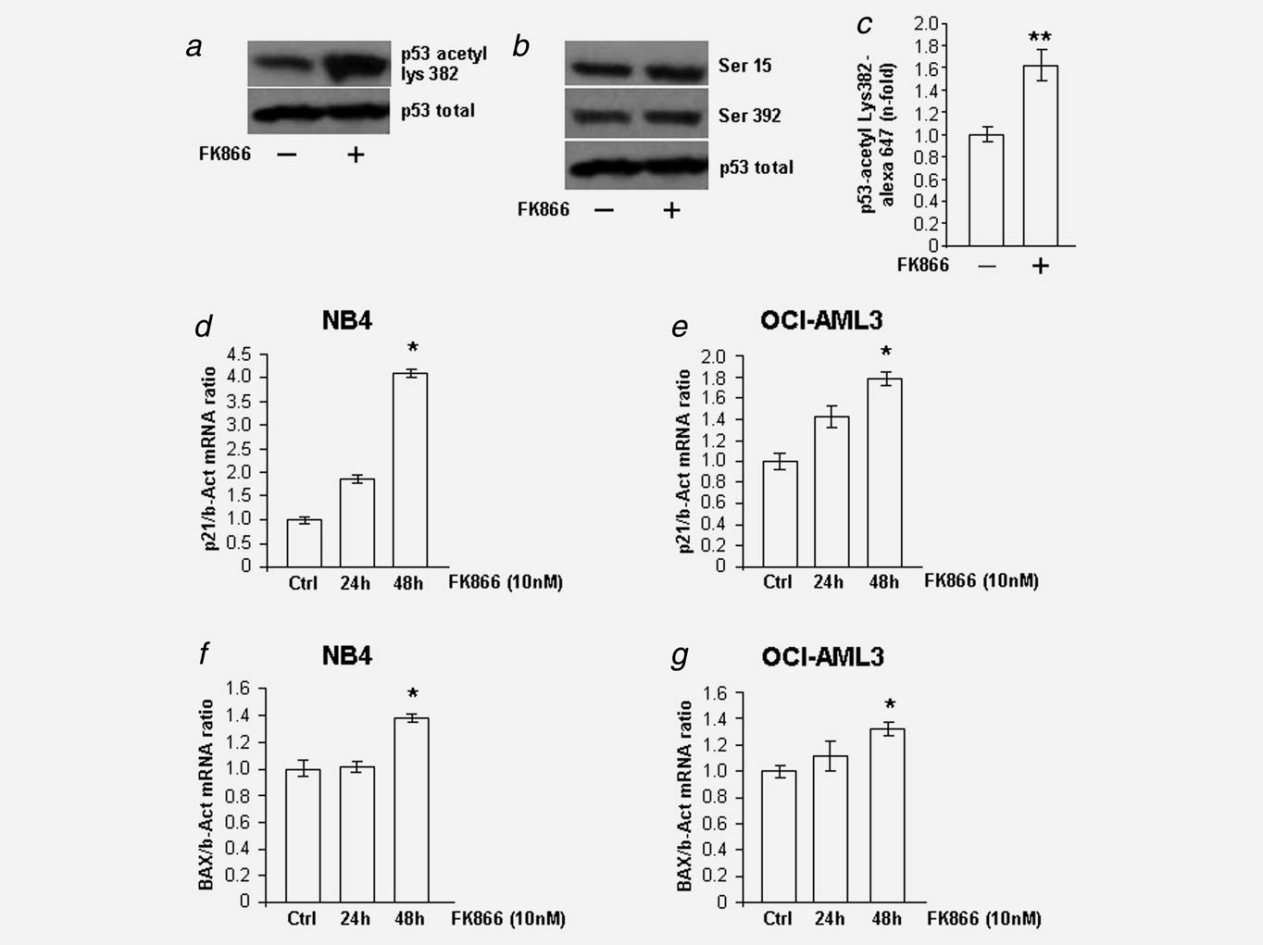
Cells exposed to FK866 display increased acetylation of p53 and elevated mRNA levels of p21 and BAX. Cells were treated with 10 nM FK866 for 48 hr. (*a*) Acetylation status at p53 lysine residue 382 in NB-4 cells was investigated in Western blot using p53 acetyl lysine 382 antibody. (*b*) Phosphorylation status of p53 residues serine 15 and serine 392 in NB-4 cells was investigated by Western blot using specific antibodies. The level of total p53 was used as the loading control. (*c*) After incubation with FK866 the OCI-AML3 cells were (*b*) intracellularly stained with Alexa Fluor 647 mouse anti-p53 K382 acetyl. Afterwards, the stained cells were subjected to flow cytometry analysis. (*d*) Following this, the mRNA expression level of p53 target gene *p21^WAF1/CIP1^* in NB-4 cells and (*e*) in OCI-AML3 cells was quantified by qRT PCR. (*f*) The mRNA expression level of p53 target gene *BAX* was also quantified in NB-4 cells and (*g*) in OCI-AML3.

To further address the mechanism of FK866-induced cell cycle arrest and apoptosis, we examined the effect of FK866 on the acetylation status of p53 in NB-4 and OCI-AML3 cell lines. At least eight lysine residues in p53 protein are known to be acetylated but the *in vivo* relevance of p53 acetylation at these residues is largely unclear.^14^ Previous studies suggest that in the presence of different extracellular stresses, acetylation of p53 at multiple lysine residues might help in a better co-ordination of p53-mediated downstream signaling.^26^–^29^ Since SIRT1-mediated inhibition of p53 functions involves mainly the deacetylation at lysine 382,^8^, ^9^, ^30^ and FK866 targets SIRT1 by inhibition of NAMPT/NAD^+^ pathway, we were interested to examine the influence of FK866 on the acetylation of p53 at lysine 382. We observed that the acetylation levels of p53 were strongly increased in NB-4 cells treated with FK866 (Fig. 4*a*). To demonstrate that treatment with FK866 does not influence the phosphorylation status of p53, we performed immunoblot studies using specific antibodies against phosphoserine 15 and phosphorserine 392 of p53. The results revealed (Fig. 4*b*) that FK866 has no influence on the phosphorylation levels of p53 at these sites. Although OCI-AML3 cells express WT p53, our attempts to demonstrate the influence of FK866 on acetylation of p53 at lysine 382 by immunoblotting approach were unsuccessful due to undetectable levels of p53. In the case of OCI-AML3 cells, we therefore made use of flow cytometry analysis to detect endogenous acetylation level of p53 at lysine 382, as this method is more sensitive compared to immunoblotting. For that, we performed intracellular staining of endogenous acetylated p53 using mouse anti-p53 K382 acetyl labeled with Alexa Fluor 647. We could successfully detect the acetylated p53 protein in OCI-AML3 cells and found that the acetylation at lysine 382 residue was modestly increased upon FK866 treatment (Fig. 4*c*). Our notion is further supported by similar results obtained in HEK293T cell line where p53 is detectable under basal conditions in Western blot. We observed that p53 was one among the several proteins whose acetylation was strongly increased when cells were treated with FK866.^31^

Further, we measured the mRNA expression of p21, involved in cell cycle control, and BAX, involved in apoptosis, in NB-4 and OCI-AML3 cell lines.^32^–^35^ The mRNA expression level of both p21 (Figs. 4*c* and 4*d*) and BAX (Figs. 4*e* and 4*f*) was increased at 48 hr after treatment with FK866 in both the cell lines. These results indicate that p53 acetylation is involved downstream of FK866-mediated signaling in regulating the expression of *p21* and *BA*X in both NB-4 and OCI-AML3 cells.

### Inhibition of sirtuins by nicotinamide imposes cytotoxic effects on both NB-4, OCI-AML3 and MOLM-13 leukemia cell lines

**Figure 5:**
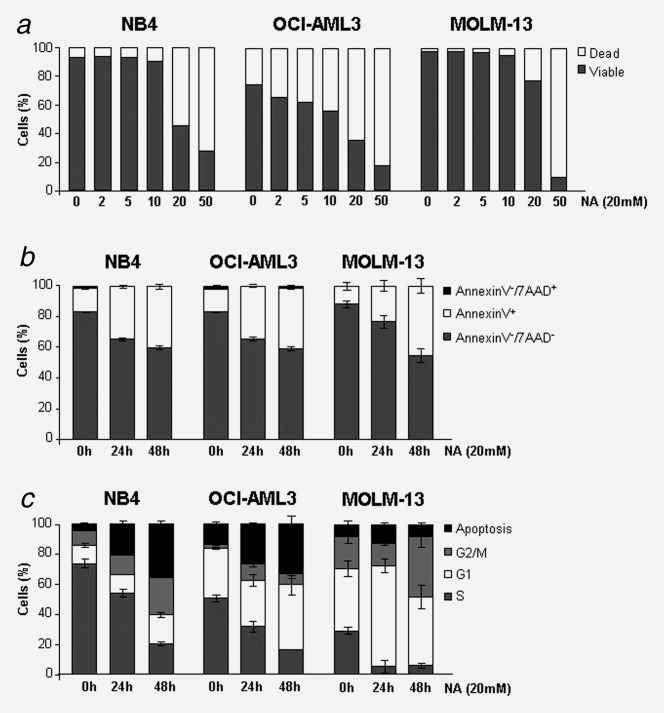
Administration of NA increases cell cycle arrest and apoptosis of responding cell lines. Cells were treated with the indicated concentrations of NA for different time points. (*a*) After treatment of NB-4 cells, OCI-AML3 cells and MOLM-13 cells with the indicated concentrations of NA for 48 hr, percentage of viable and apoptotic/dead fractions were determined using 7-AAD staining in combination with combined changes of cellular FSC *vs*. SSC dot plot as previously described.^35^ (*b*) Percentage of AnnexinV^−^/7AAD^−^ (live), AnnexinV^+^ (apoptotic), AnnexinV^−^/7AAD^+^ (necrotic) cells was measured using flow cytometry in cell lines NB-4, OCI-AML3 and MOLM-13. (*c*) BrdU/7-AAD cell cycle analysis of NB-4 cells, OCI-AML3 cells and MOLM-13 cells. Diagram shows percentage of cells in apoptosis, G2/M-, G1- or S-phase.

Among all the SIRTs, SIRT1 is the major player in deacetylation of p53. Therefore, we performed knockdown of SIRT1 and analyzed its influence on cell cycle and apoptosis. Knockdown of SIRT1 in NB-4 cells had no observable changes in apoptosis and cell cycle arrest (data not shown). NA is a non-competitive inhibitor of SIRTs at high concentrations and has been recently shown to be highly effective in killing CML cells through specific activation of the p53 pathway.^16^ Based on our observation that the sensitivity of NB-4, OCI-AML3 and MOLM-13 cells toward NA begins at 20 mM concentration (Fig. 5*a*), we used 20 mM of NA for our further experiments. We found that treatment with NA resulted in increased apoptosis (Fig. 5*b*) and decreased cell count in S-phase (Fig. 5*c*). These results suggest that inhibition of SIRTs plays a critical role in the killing of leukemic cells and justifies the importance of NAMPT/NAD^+^ pathway in maintaining the status of leukemic cells.

### Role of p53 in FK866-mediated increase in apoptosis and cell cycle arrest

*BAX* and *p21* are well known target genes of p53. Activation of p53 has been shown to be mirrored by increased expression of these genes.^32^–^35^ To check the direct influence of p53 on the expression of the target genes *p21* and *BAX*, we performed shRNA-mediated knockdown of p53 in NB-4 (Supporting Information Fig. 3A), OCI-AML3 (Supporting Information Fig. 3D) and MOLM-13 (Supporting Information Fig. 3G) cells. We observed significant decrease in the expression of p21 and BAX in NB-4 (Supporting Information Figs. 3B and 3C) and OCI-AML3 (Supporting Information Figs. 3E and 3F) and MOLM-13 (Supporting Information Figs. 3H and 3I) cells after successful knockdown of p53. Next, we measured NAD^+^ as well as ATP concentrations and analyzed apoptosis of cells in S-phase in p53 knocked down cells. The NAD^+^ content in OCI-AML3 cell line was increased (Supporting Information Fig.4C) after p53 knockdown with no change in ATP content (Supporting Information Fig. 4D). No significant change in the NAD^+^ or ATP content was observed in NB-4 cells after knockdown of p53 (Supporting Information Figs. 4A and 4B). Under basal conditions, knockdown of p53 resulted in decreased apoptosis and increased cell numbers in S-phase in OCI-AML3 (Supporting Information Figs. 5C and 5D) and MOLM-13 cell lines (Supporting Information Figs. 5E and 5F). In contrast, knockdown of p53 in NB-4 cells had no significant influence on apoptosis (Supporting Information Fig. 5A) and cell cycle profile (Supporting Information Fig. 5B), indicating that under basal conditions mutant p53 (C176F) is inactive to execute its tumor suppressor functions but the treatment with FK866 triggers the acetylation of p53 and restores its activity in NB-4 cells.

**Figure 6:**
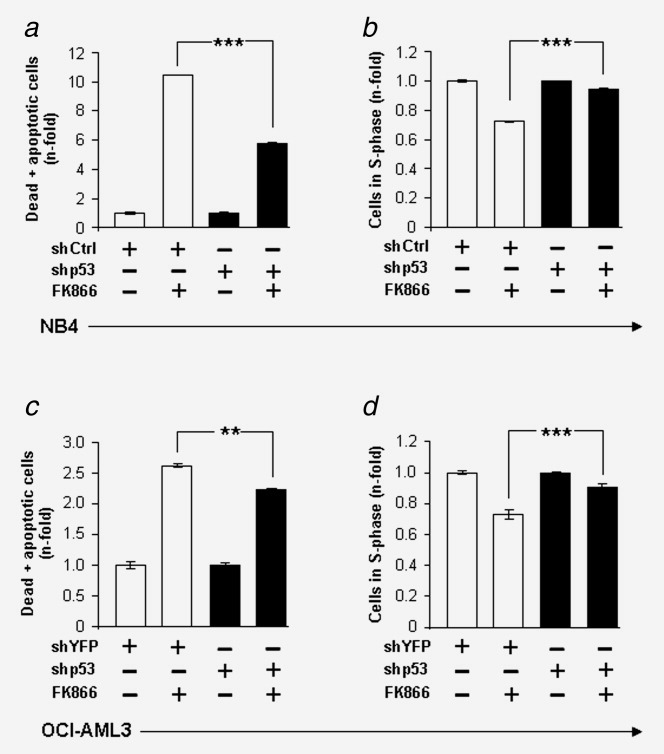
Knockdown of p53 attenuates the cytotoxic effects imposed by FK866. Control transduced (shCtrl/shYFP) or shp53 transduced cells were either untreated or treated with 10 nM FK866 for 72 hr. (*a*) The *n*-fold change of NB-4 cells and (*c*) OCI-AML3 cells, with and without knockdown of p53, undergoing apoptosis upon exposure to FK866 was determined on the basis of Annexin V staining. (*b*) The *n*-fold change of cells in S-Phase in NB-4 cell populations and (*d*) OCI-AML3 cell populations, with and without knockdown of p53, upon exposure to FK866 was determined on the basis of BrdU/7-AAD cell cycle analysis.

To check whether p53 plays a direct role in executing the FK866-mediated cytotoxic effect, we measured apoptosis and cell cycle arrest in the absence or presence of FK866 in NB-4 and OCI-AML3 cells after knockdown of p53, and compared it with control cells. Knockdown of p53 attenuated significantly the apoptosis and cell cycle arrest imposed by FK866 in both NB-4 (Figs. 6*a* and 6*b*) and OCI-AML3 (Figs. 6*c* and 6*d*) leukemic cell lines.

## Discussion

In the current study, we provide evidence for the involvement of p53 in the cytotoxic effects imposed by FK866 on myeloid leukemia cell lines. This is based on three lines of evidence; (*i*) FK866 increases the levels of acetylated p53, the functionally active form of p53, by inhibition of NAMPT/NAD^+^/SIRT pathway, (*ii*) by this mechanism FK866 increases expression of *p21* and *BAX*, genes relevant in p53-mediated tumor suppressor functions and (*iii*) in the absence of functional p53, the effect of FK866 on leukemia cells is attenuated. The resistance of cancer cells, including leukemic cells, to existing chemotherapy is considered to be a challenging task in the treatment options. Identification and characterization of factors causing refractory AML suggests that several mechanisms of MDR (multi drug resistance) exist in AML. Recently, in cases of AML, mutation in p53 gene was shown to be associated with drug resistance and increased mortality. The fact that mutation of p53 is a rare event in AML raises the possibility that drug resistance in cases of AML, containing wild type p53, might be due to post-translational inactivation of p53 by overexpression of negative regulators like, MDM2 or SIRTs. Functional activation of p53 by inhibition of negative regulators in leukemic cells is a useful approach to enhance the cytotoxicity of existing chemotherapeutic agents.^13^, ^16^ In addition, restoring wild type functions to mutant p53 by using small molecule inhibitors has proven to be an effective strategy to induce apoptosis in cancer cells.^37^, ^38^ Our results obtained in the NB-4 cell line indicate that FK866-mediated acetylation of mutant p53 restores its ability to execute tumor suppressive functions. The finding has a broad implication in improving the clinical efficacy of FK866. Categorizing AML patients on the basis of p53 status (WT p53, mutant p53 whose function can be restored by the use of FK866 or loss/truncation of p53 protein) will be indeed useful in characterizing which patients will be most benefited by FK866 treatment.

Normal cells posses a limited pool of NAD^+^ whereas cancer cells have high rate of NAD^+^ turnover. This is due to the fact that cancer cells have a high metabolic rate and elevated ADP-ribosylation activity, predominantly mediated by the poly(ADP-ribose) polymerases (PARPs).^39^, ^40^ One mechanism by which cancer cells maintain the required pool of NAD^+^ is by overexpression of the enzyme NAMPT, which is involved in NAD^+^ biosynthesis.^41^, ^42^ High NAD^+^ can be associated with activated levels of SIRT which in turn lead to inactivation of p53, as observed in several types of cancer, including leukemia.^16^, ^43^, ^44^ Our own previous findings suggest that overexpression of SIRT1 by activation of NAMPT/NAD^+^ pathway might be a prognostic factor in leukemic transformation secondary to severe congenital neutropenia.^45^ Similarly, in context to aging, it has been shown that overexpression of NAMPT delayed senescence and lengthened life span of smooth muscles cells, possibly by the p53 acetylation pathway.^46^ We observed that either inhibition of NAMPT or direct inhibition of SIRTs have similar effects on cell cycle and apoptosis in AML cells. This highlights the significance of SIRT inhibition as a potential strategy to specifically target AML cells. Our results are supported by the recent findings that inhibition of SIRT1 by NA induces cytotoxic effects on CML cells in p53 acetylation dependent manner.^16^ Intriguingly, the specificity of NA on SIRT1 is quite unclear due to its dual ability to either activate or inhibit SIRT1, depending on the dose and context.^45^, ^47^ Despite its use in the clinical trials as a therapy for cancers,^48^ further detailed investigation of its effects at molecular level is required. Therefore, our current study raises a possibility that inhibition of NAMPT by FK866 might be a better strategy to specifically target AML cells.

Inhibitors, blocking the activity of HDACs, exert a global effect on the acetylation profile of several cellular proteins.^49^ Therefore, the inhibition of NAD^+^ dependent deacetylation by FK866 might also influence the function of several other proteins by modulating their acetylation status. Our initial attempt to analyze the acetylation pattern of total cellular protein in Western blot using an antibody specific for acetyl lysine revealed that the acetylation level of several proteins ranging from 20 kDa to 150 kDa was increased in the lysate extracted from NB-4 cells treated with FK866 (Supporting Information Fig. 6). Our observation that knockdown of p53 does not completely abrogate the effects of FK866 on cell proliferation and apoptosis (Fig. 5) raises possibilities that FK866 can either trigger pathways which are independent of p53 or low levels of p53 present in knockdown cells is sufficient enough to mediate FK866-mediated effects in leukemic cells. Our preliminary study suggests that FK866-mediated apoptosis in NB-4 cells might involve acetylation dependent de-phosphorylation of AKT.^22^ However the current study is the first report for the evidence that FK866 induces apoptosis in leukemic cells, including NB-4 cells, by catalyzing the functional activity of p53. Further, identification of novel p53 dependent and independent targets involved in FK866-mediated signaling in leukemic cells is the subject of our future investigation.

In summary, our current study highlights the importance of the p53 pathway downstream of FK866 in the death of leukemic cells. The ability of FK866 to activate the tumor suppressor functions of p53 in leukemic cells, harboring WT or mutant p53 may provide a rationale for the consideration of FK866 in combination with existing chemotherapeutic agents, in the treatment of leukemia with functional p53.

## Supplementary material

Supporting Information Figure 1. Percentage of Kasumi cells undergoing apoptosis and cell cycle arrest increases upon exposure to FK866. Cells were treated with 10 nM FK866 for indicated time points. (A) BrdU/7-AAD cell cycle analysis of control cells and treated cells. Diagram shows percentage of cells in apoptosis, G2/M, G1 or S-phase. (B) Percentage of annexinV positive cells indicating apoptosis compared to viable cells was measured using flow cytometry.

Supporting Information Figure 2. FK866 mediated impact on NAD+ and ATP levels of non-responding cell lines. Cells were treated with 10 nM FK866 for indicated time points. (A) NAD+ levels were measured after exposure to FK866 in Kasumi cells and in K562 cells (B) ATP levels were measured after exposure to FK866 in Kasumi cells and in K562 cells.

Supporting Information Figure 3. Effect of p53 knock down on p53 target gene mRNA expression. Level of mRNA expression was quantified via qRT PCR after lentiviral knock down of p53. (A) Confirmation of p53 knockdown in NB-4 cells, (D) in OCI-AML3 cells and (G) MOLM-13 cells. (B) Level of mRNA expression of p53 target gene p21WAF1/CIP1 in NB-4 cells, (E) in OCI-AML3 cells and (H) MOLM-13 cells. (C) Level of mRNA expression of p53 target gene BAX in NB-4 cells, (F) in OCI-AML3 cells and (I) MOLM-13 cells.

Supporting Information Figure 4. Impact of p53 knock down on NAD+ and ATP levels of responding cell lines. (A) NAD+ levels were measured after NB-4 cells and (C) in OCI-AML3 cells with or with out p53 knockdown. (B) ATP levels were measured in NB-4 cells and (D) in OCI-AML3 cells with or with out p53 knockdown.

Supporting Information Figure 5. Influence of p53 knock down on apoptosis and cell cycle of responding cell lines. (A) Percentage of annexinV+ (apoptotic) cells was measured using flow cytometry in the cell lines NB-4, (C) in OCI-AML3 and (E) in MOLM-13. (B) BrdU/7-AAD cell cycle analysis of NB-4 cells, (D) of OCI-AML3 cells and (F) of MOLM-13 cells. Diagram shows percentage of cells in S-phase.

Supporting Information Figure 6. Cells exposed to FK866 display increased acetylation levels of various proteins. Cells were treated with 10 nM of FK866 for indicated time points. (A) Western blot was performed using acetyl lysine antibody. Left: high exposure; right: low exposure of x-ray film. Loading control is represented by unspecific bands of the acetyl lysine antibody.
