# Supplementary figures and images for: Involvement of p53 in the cytotoxic activity of the NAMPT inhibitor FK866 in myeloid leukemic cells
Source: Int J Cancer. 2012 Jul 20;132(4):766–74. doi: 10.1002/ijc.27726 (PMC3562481; doi:10.1002/ijc.27726)

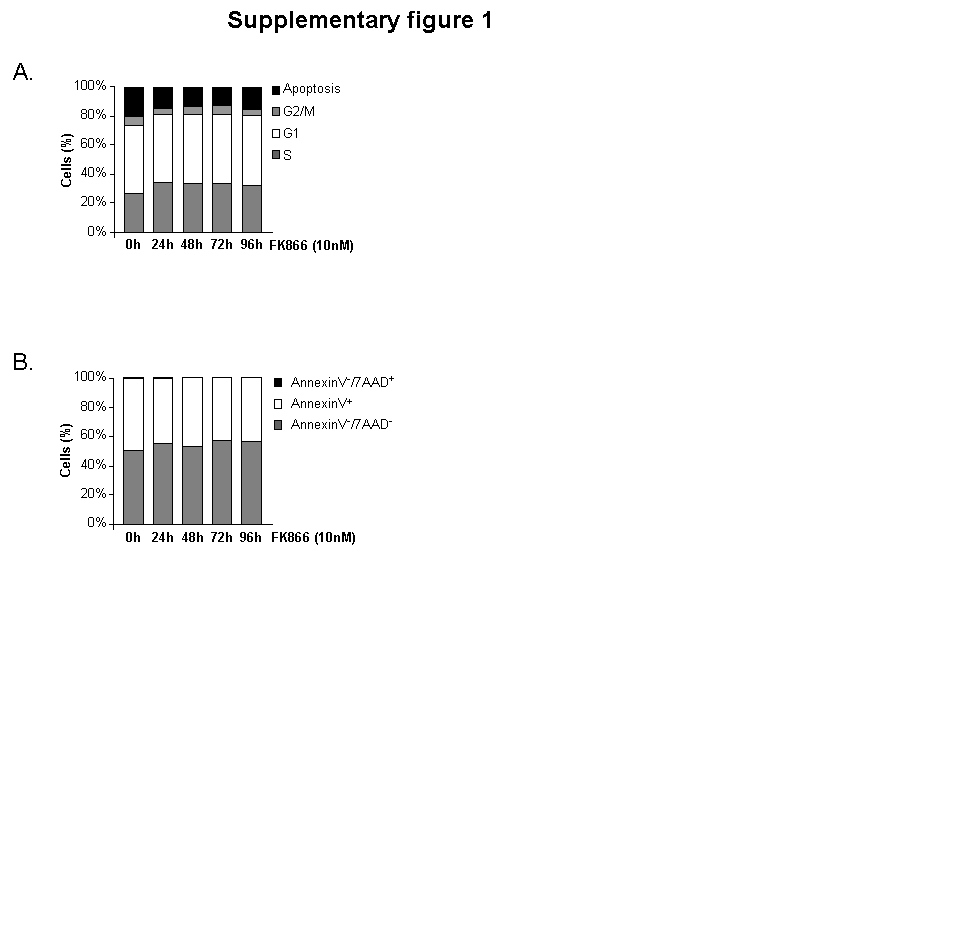

Supplement: Supplementary file 1 [file ijc0132-0766-SD1.tif]

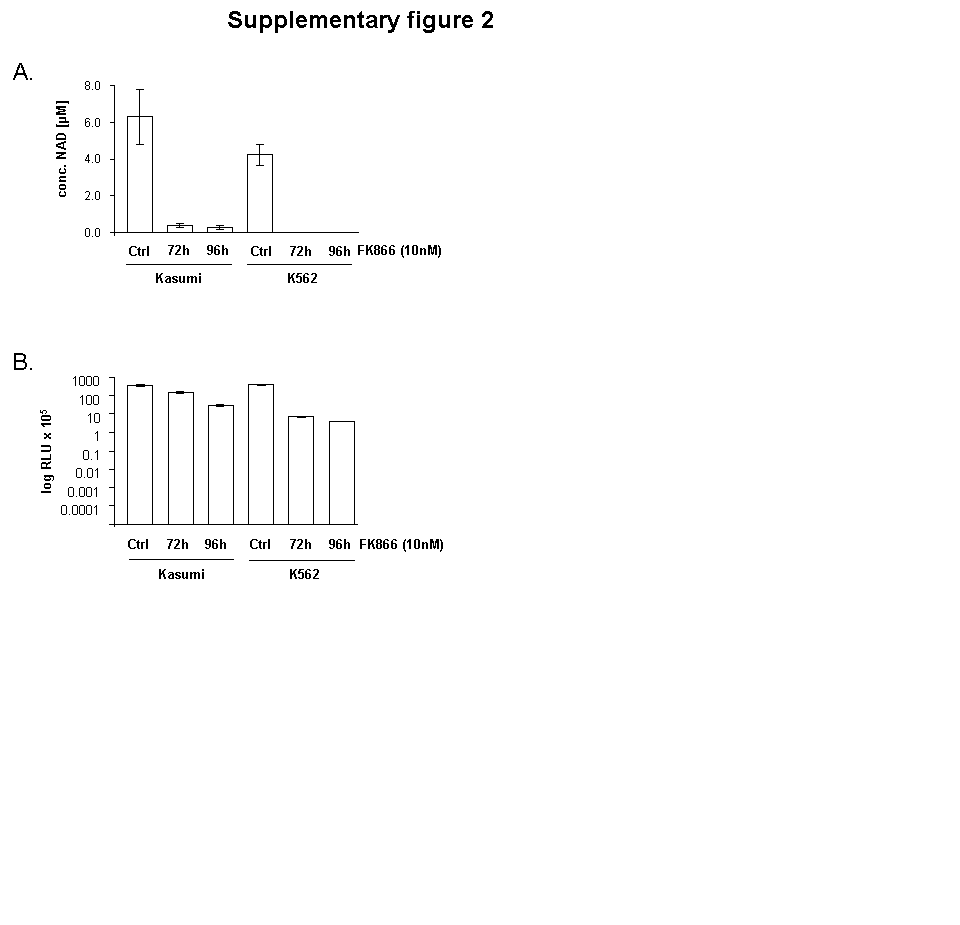

Supplement: Supplementary file 2 [file ijc0132-0766-SD2.tif]

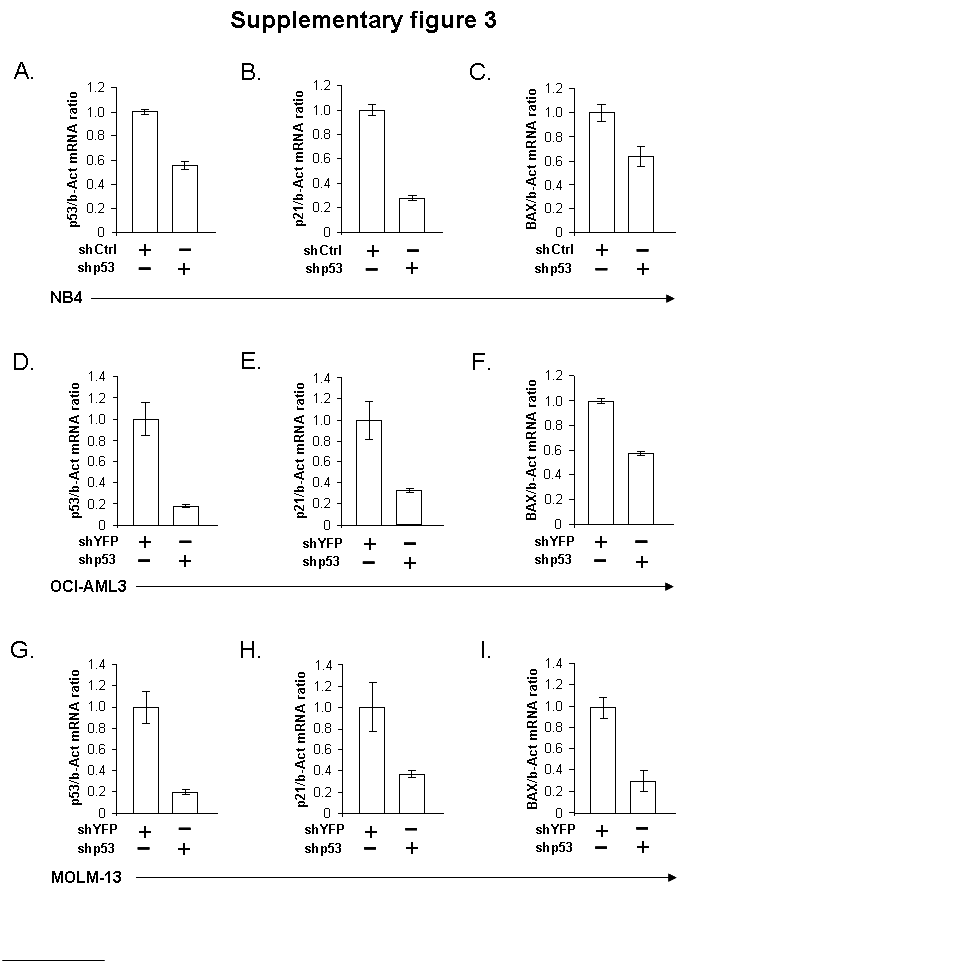

Supplement: Supplementary file 3 [file ijc0132-0766-SD3.tif]

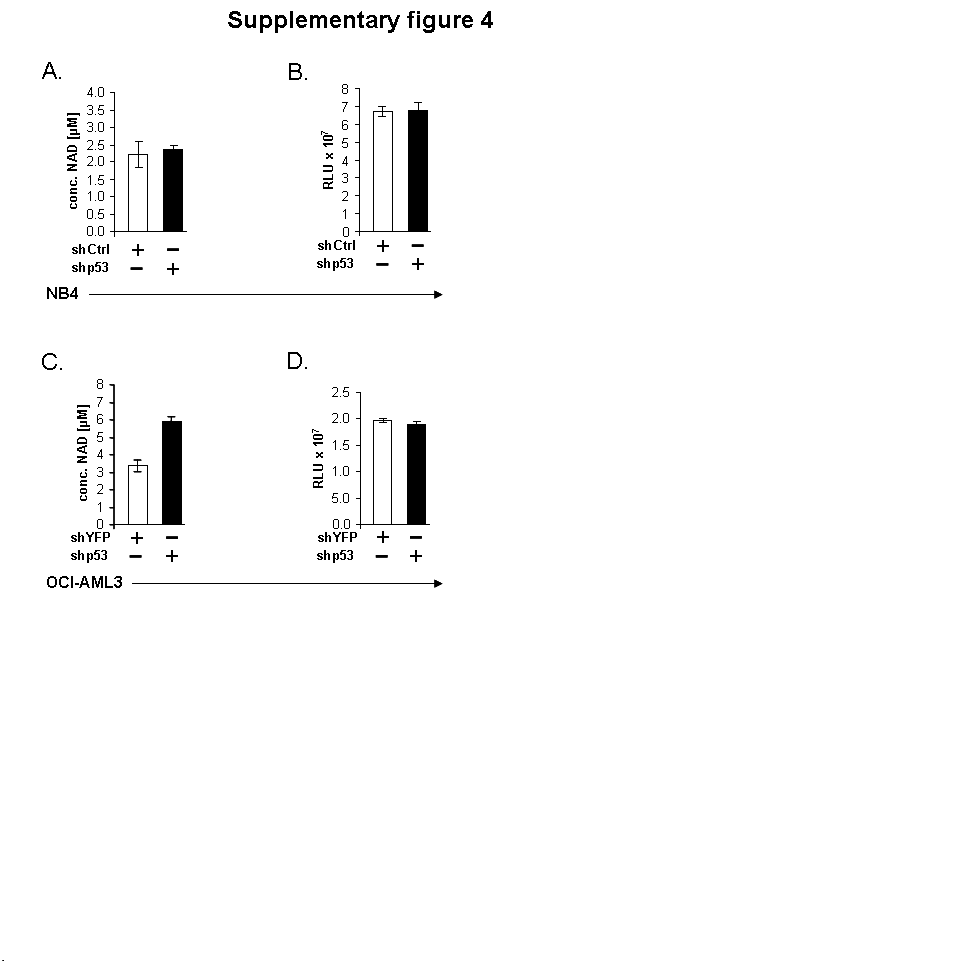

Supplement: Supplementary file 4 [file ijc0132-0766-SD4.tif]

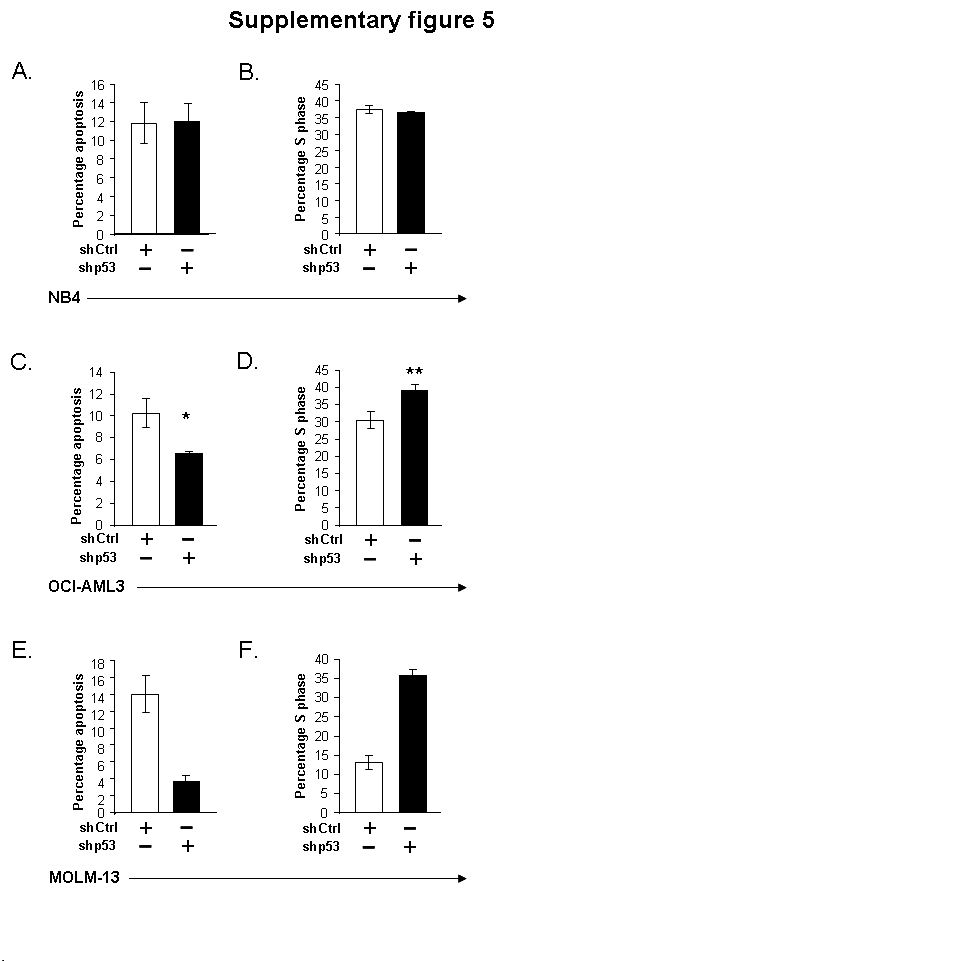

Supplement: Supplementary file 5 [file ijc0132-0766-SD5.tif]

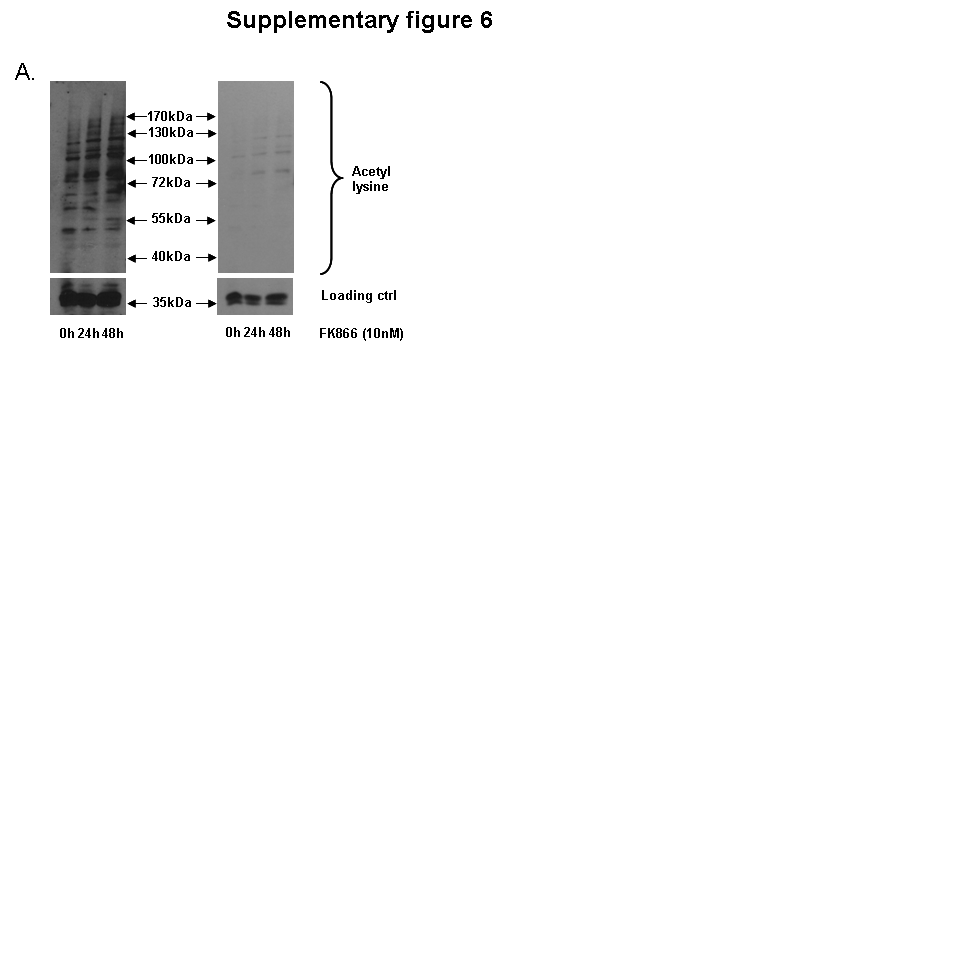

Supplement: Supplementary file 6 [file ijc0132-0766-SD6.tif]
